# The mental health and substance misuse needs of male ex-armed forces personnel in prison

**DOI:** 10.1080/14789949.2017.1352012

**Published:** 2017-07-12

**Authors:** Verity Wainwright, Charlotte Lennox, Sharon McDonnell, Jenny Shaw, Jane Senior

**Affiliations:** a Centre for Mental Health and Safety, University of Manchester, UK

**Keywords:** Ex-armed forces personnel, forensic mental health, substance misuse, prison

## Abstract

Ex-armed forces personnel constitute the largest known occupational group in prison but there is little evidence regarding their mental health, or substance misuse, needs. A total of 105 participants were interviewed and measures assessing symptoms of common mental health (CMH) problems and substance misuse were completed along with a review of their health care records. Forty (38%) participants screened for current CMH problems (CCMH) and high levels of dual symptomology and alcohol misuse were assessed. Thirty-nine (37%) had a mental health diagnosis recorded, most commonly for post-traumatic stress disorder (PTSD), depression and personality disorder. Those who screened for a CCMH problem were more likely to have pre-service vulnerability to negative health outcomes and those with dual symptomology were more likely to have experienced deployment during their service. Findings suggest the mental health needs of this group are similar to the general prison population. Potentially higher prevalences of PTSD and alcohol misuse may direct service provision.

## Introduction

The high prevalence of mental health problems in the prison population worldwide is well-documented (Fazel & Danesh, 2002; Singleton, Meltzer, & Gatward, 1998). However, despite former armed service personnel constituting the largest known occupational group in prisons in England and Wales (Howard League, 2011), little is known about their mental health and substance misuse needs and whether these differ to prisoners without a service history. In a sample of 315 ex-armed forces personnel in the community, Iversen et al. (2005) found that the most common diagnoses were for depressive episodes and anxiety disorders. Current evidence also suggests CMH problems may be more prevalent in incarcerated ex-armed forces personnel compared to the general prison population (Phillips, 2014). The Howard League inquiry into former armed service personnel in the criminal justice system (CJS) (2011) highlighted alcohol misuse as a concern, emphasising its role in relation to both mental health problems and offending behaviour. Similarly, Her Majesty’s Inspectorate of Prisons (2014) reported that ex-service personnel were more likely to present as depressed and/or suicidal on arrival into prison than the general prison population (although the military service of participants was not verified).

Previous studies have investigated the aetiology of mental health problems amongst armed forces personnel, examining the influence of pre-service factors, military experiences and post-service issues. Studies have shown that CMH problems and alcohol misuse are the most frequent difficulties encountered by personnel returning from deployment (Fear et al., 2010; Hotopf et al., 2006). Fear et al. (2010) reported prevalence rates of 20% for CMH problems, 13% for alcohol misuse and 4% for probable PTSD. Deployment was also significantly associated with alcohol misuse, and combat personnel were more likely to report probable PTSD (Fear et al., 2010). Some groups within the armed forces tend to fare worse with regard to their mental health than others. Deployed reservist personnel have consistently been found to be more likely to report probable PTSD than regular personnel (Fear et al., 2010; Hotopf et al., 2006). Furthermore, early service leavers (ESLs; those who have served for four years or less) were more likely to report symptoms of common mental disorders, probable PTSD, fatigue and multiple physical symptoms compared to other service leavers (Buckman et al., 2012).

However, the link between pre-enlistment and post-service factors has also been discussed in relation to the mental health of armed forces personnel. Pre-enlistment vulnerability (such as poor family relationships and fighting at school) has been found to be an important determinant of the mental health of serving personnel. Using questionnaire data from a large sample of UK regular armed forces personnel (*n* = 7937), Iversen et al. (2007) found pre-enlistment vulnerability was associated with a number of negative health outcomes including general psychological ill health, PTSD and alcohol use. Pre-enlistment vulnerability was also associated with having served in the Army in a lower rank, being single and having low educational attainment (Iversen et al., 2007). Considering post-service, whilst the majority of personnel make the transition from military to civilian life successfully, it is accepted as a problematic time (Howard League, 2011). Potential issues include relationship difficulties, family breakdown, unemployment, homelessness and potential loss of purpose/identity previously provided by employment in the armed forces. One particular concern is the loss of the ‘family’ of the armed forces and the social isolation felt as a result. Hatch et al. (2013) found that service leavers reported more social isolation than serving personnel and that less social activity and smaller social networks, were associated with symptoms of common mental disorder and PTSD. Being in relationship was found to be a protective factor (Hatch et al., 2013).

Improving our understanding of the mental health needs of ex-armed forces personnel in prison is imperative if appropriate support can be provided to them both in prison, and on release. To this end, this study aimed to identify the mental health and substance misuse needs of a sample of male ex-armed forces personnel in prison. In order to explore the potential aetiology of these problems for this group, the study also aimed to compare characteristics between groups of those who screened positively for CCMH problems, substance misuse, and those with dual symptomology.

## Method

This study formed part of a larger mixed methods study exploring the pathways to offending and mental health needs of male ex-armed forces personnel in prison.

### Sample and procedure

The study was conducted between February 2014 and July 2015 in six adult male prison establishments in England. A range of local, training and open establishments were included that all routinely asked the question regarding previous military service and recorded this information. All known ex-armed forces personnel residing in the establishments when the study began, and all new receptions into custody during the study period who identified as having previously served in the armed forces, were approached to take part. Fourteen prisoners declined to take part resulting in a sample size of 105. The Veterans in Custody Support Officer (VICSO) in each establishment made the initial approach to all participants and verified their service. Participants were interviewed by the first author in a private room within the prison. The health care notes of each participant were also reviewed to capture any recorded mental health diagnoses. All participants gave written, informed, consent to take part in the study.

Demographic and background information, military experience and circumstances post-armed forces were collected for all participants via a researcher administered questionnaire. As part of the questionnaire, participants were screened for pre-service vulnerability to negative health outcomes. The same measure used by Iversen et al. (2007) in their study of the influence of childhood adversity on health outcomes amongst a sample of UK military personnel was utilised. The measure includes sixteen items of protective and adverse factors, with questions in three domains: family relationships, parenting and adolescent behaviour. Those who reported four or more adverse factors were considered to have pre-service vulnerability. In addition, the following standardised assessments were administered to assess the participants’ mental health and substance use:

(a) General Health Questionnaire-12 items (GHQ-12) (Goldberg, 1972) was used as a measure of current mental health. The GHQ-12 focuses on two major areas: the inability to carry out normal functions and the appearance of new and distressing experiences. Items are rated using the standard dichotomous scoring style (0, 0, 1, 1) and scores range between 0 and 12. A cut-off score of 3 is generally used to establish ‘caseness’ in UK populations (Hassan et al., 2011). However, previous studies have identified that a higher cut-off is more appropriate to discriminate the well-being of those in prison (Andersen, Sestoft, Lillebæk, Gabrielsen, & Hemmingsen, 2002; Hassan et al., 2011). Therefore, a cut-off score of 7 was adopted to determine prison caseness in this study.
(b) Patient Health Questionnaire-9 (PHQ-9) (Kroenke & Spitzer, 2002) a nine-item scale that screens for, and assesses the severity of, symptoms of depression. This scale scores each of the 9 Diagnostic and Statistical Manual of Mental Disorders (DSM-IV) criteria on a four-point scale (0, 1, 2, 3) and scores range between 0 and 27. The National Health Service (NHS) Improving Access to Psychological Therapies (IAPT) cut-off score of 10 was used to determine caseness.
(c) Generalised Anxiety Disorder-7 (GAD-7) (Spitzer, Kroenke, Williams, & Lowe, 2006) a seven-item scale used to screen for Generalised Anxiety Disorder. Scores range between 0 and 21 with each item scored on a four-point scale. The NHS IAPT cut-off score of 8 was used to determine caseness.
(d) PTSD Checklist-Civilian (PCL-C) (Blanchard, Jones-Alexander, Buckley, & Forneris, 1996) measure enables screening for symptoms of PTSD using DSM-IV criteria. The PCL-C is the civilian version of the checklist and asks about symptoms in relation to ‘stressful experiences’. The measure was chosen as it encompasses all stressful experiences and is not limited to military experiences. The measure has 17 items scored on a five-point scale (1, 2, 3, 4, 5). Scores range 17–85. A cut-off score of 40 was used to determine caseness to screen for all cases of possible PTSD (Blanchard et al., 1996; Rona et al., 2014).
(e) AUDIT: Alcohol Use Disorders Identification Test (Babor, Higgins-Biddle, Saunders, & Monteiro, 2001) comprises ten questions addressing four areas: alcohol intake; abnormal drinking behaviour and alcohol dependence; the link between alcohol consumption and the detection of psychological effect; and alcohol-related problems. A five-point scale (0, 1, 2, 3, 4) is used to score the measure and scores range between 0 and 40. The measure uses a cut-off score of 8 to screen for hazardous drinking and 16 to determine harmful or dependent drinking (Babor et al., 2001). Therefore, in this study a score of 8 was considered to demonstrate harmful and/or hazardous drinking and was used to determine caseness.
(f) The Drug Abuse Screening Test (DAST) (Skinner, 1982) a twenty-eight item ‘yes’ or ‘no’ scale that yields a quantitative index of the range of problems associated with drug abuse. ‘Yes’ answers are given a score of 1 and ‘no’ answers a score of 0, with the exception of three reverse scored items. Scores range 0–28. A score of more than 6 is considered to demonstrate possible substance misuse disorder (Skinner, 1982) and was therefore used to determine caseness.

### Statistical analysis

Data were analysed using SPSS (Statistical Package for the Social Sciences) for Windows version 22 (IBM Corp, 2013). Descriptive statistics and frequencies are reported to describe the characteristics of the sample. Those who screened positively for CCMH problems (i.e. met the ‘caseness’ cut-off score for one or more of the PHQ-9, GAD-7 and PCL-C measures) were compared with those who did not. Likewise, those who screened positively for an alcohol or drug misuse problem using the AUDIT and DAST measures appropriate cut-off scores were compared with those who did not. Comparisons were also made between those who demonstrated dual symptomology (i.e. screened positive for a CCMH problem and an alcohol or drug misuse problem) and those who did not.

To explore differences between groups, they were compared on a number of variables: age; length of service; whether or not participants reported low levels of social contact (regular contact with less than three people); whether or not they reported having financial problems prior to prison; employment status prior to prison; experience of deployment; and pre-service vulnerability. In addition, composite variables were developed to compare marital status (those reporting single status compared to those who were married or in a long-term relationship) and rank (private rank compared to higher ranks). Independent samples *t*-tests and the chi-squared statistic were used to identify any significant mean score differences between groups (degrees of freedom are reported for *t*-test comparisons and odds ratios for chi squared analyses).

### Ethics approval and consent to participate

The study was granted National Health Service (NHS) approval from a research ethics committee (13/WA/0332) as well as National Offender Management Service (NOMS) approval (2014-208). The governor, and health care provider, at each prison site also approved the study. All participants provided informed, written, consent to take part in the study.

## Results

In the 18-month study period, 105 male ex-armed forces personnel in prison were recruited to the study. The mean age of the sample was 42 (SD = 14; range 20–88 years) comprising 88% of the total eligible population (*n* = 119). Most were of white ethnicity (*n* = 100, 95%). Socio-demographic characteristics of the sample are presented in Table 1.

**Table 1.** Socio-demographic characteristics of the sample (*n* = 105).

Characteristic	*n* (%)
Education level	
GCSEs or equivalent	63 (60)
Left school with no qualifications	27 (26)
A levels or equivalent	9 (9)
Degree or equivalent	3 (3)
Professional qualifications	3 (3)
Marital status	
Single	46 (44)
Married	19 (18)
Long-term relationship	18 (17)
Divorced/separated	18 (17)
Widowed	4 (4)
Employment status prior to prison	
Employed	60 (57)
Unemployed	37 (35)
Retired	7 (7)
Disabled/sickness benefit	1 (1)
Living circumstances prior to prison	
Rented property	45 (43)
With friends or family	29 (28)
Own home	20 (19)
Homeless	4 (4)
Other	4 (4)
Hostel/B&B	3 (3)

### Armed forces service

The majority of the sample had served as a Regular (*n* = 91, 87%) rather than as a Reserve. The vast majority had served in the Army (*n* = 85, 81%), followed by the Navy (*n* = 15, 14%) and the Royal Air Force (RAF) (*n* = 5, 5%). The mean age of participants at enlistment was 18 (SD = 3; range 15–29 years). Participants had served on average 6 years (SD = 5; range 1–30 years). The most common rank of participants (using the Army ranking system) was Private (*n* = 76, 72%), primarily serving in a combat role (*n* = 70, 67%). Most of the sample had experienced deployment (*n* = 64, 61%) and the mean number of deployments was 2 (SD = 2; range 1–9). The most common method of leaving the armed forces was via standard end of service contract (*n* = 46, 44%), followed by premature voluntary release or sign-off (*n* = 19, 18%) and disciplinary discharge (*n* = 19, 18%). Nine (9%) had been medically discharged.

### Mental health

Forty-three (41%) participants self-reported having a CMH problem; however, of these, only 20 (47%) reported that they were currently receiving treatment in prison, and a further 8 (19%) stated they were on a waiting list. In the health care records of participants, 39 (37%) had a mental health diagnosis recorded; the most common primary diagnoses recorded were: PTSD (*n* = 17, 16%), followed by depression (*n* = 12, 11%) and personality disorder (*n* = 5, 5%). None of the participants (*n* = 105, 100%) were considered to be at risk of self-harm or suicide at the time of the file review, but 21 (20%) had previously been cared for under Assessment, Care in Custody and Treatment (ACCT) procedures during their current term in prison.

Using the measures, 37 (35%) met the cut-off for GHQ-12 prison caseness and 40 (38%) participants screened positively for a CCMH problem (i.e. screened in on one or more of the measures for depression, anxiety or PTSD as screened by the PHQ-9, GAD-7 and PCL-C, retrospectively). Regarding those who screened positively for CCMH problems, the majority screened in on one measure only (*n* = 16, 40%), 14 (35%) participants screened in on two measures and 10 (25%) participants on all three measures. Table 2 shows the mean score and the number of cases and non-cases for each measure.

**Table 2.** Screening results by measure (*n* = 105).

Measure	*n* (%)
GHQ-12 screening	
Total score mean (SD)	4.41 (2.29)
Cases	37 (35)
Non-cases	68 65)
PHQ-9 screening	
Total score mean (SD)	5.18 (6.28)
Cases	21 (20)
Non-cases	84 (80)
GAD-7 screening	
Total score mean (SD)	4.84 (5.50)
Cases	28 (27)
Non-cases	77 (73)
PCL-C screening	
Total score mean (SD)	30.29 (15.58)
Cases	26 (25)
Non-cases	79 (75)
AUDIT screening	
Total score mean (SD)	13.87 (12.10)
Cases	59 (56)
Non-cases	46 (44)
DAST	
Total score mean (SD)	4.54 (6.85)
Cases	29 (28)
Non-cases	76 (72)

The majority of participants (*n* = 71, 68%) screened as having pre-enlistment vulnerability to negative health outcomes. Those with CCMH problems were significantly more likely to demonstrate pre-service vulnerability than those who screened negatively (see Table 3). Those who screened positively for CCMH were also significantly more likely to report being single, and having had money problems and limited social contact in their lives prior to entering prison. There were no other significant associations between groups.

**Table 3.** Group comparisons (*χ*^2^ and *t*-tests) of those who screened positively for CCMH problems (*n* = 40) and those who did not (*n* = 65).

Variable	CCMH	No CCMH	OR	95% CI	*t* (d.f.)
Single marital status, *n* (%)	32 (80)	36 (55)	3.22^**^	1.29–8.06	–
Pre-service vulnerability, *n* (%)	33 (83)	38 (59)	3.35^*^	1.29–8.69	–
Private rank, *n* (%)	32 (80)	44 (68)	.134	.751–4.85	–
Experience of deployment, *n* (%)	22 (55)	42 (65)	1.21	.300–1.49	–
Unemployed prior to prison, *n* (%)	21 (53)	25 (39)	.565	.255–1.25	–
Money problems prior to prison, *n* (%)	24 (60)	16 (25)	4.59^**^	1.96–10.7	–
Low social contact, *n* (%)	17 (43)	10 (15)	.246^**^	.098–.617	–
Age, years					
Mean (SD)	40 (13.0)	43 (14.7)	–	–	1.21 (103)
Median (min–max)	37 (21–79)	42 (20–88)			
Length of service, years					
Mean (SD)	6 (4.2)	7 (5.8)	–	–	1.36 (103)
Median (min–max)	4 (1–20)	5 (1–30)			

* *p* < .05

** *p* < 0.

### Alcohol and drug misuse

Considering alcohol use in the year prior to entering prison, over half of the sample (*n* = 59, 56%) screened positively for alcohol misuse, with the majority (*n* = 40, 68%) screening positively for the more severe category of alcohol dependence. Nineteen (18%) participants reported they were currently receiving help for alcohol use in prison; this represented 32% of the population in need. Those who screened positively for alcohol misuse (Table 4) were significantly more likely to be younger, and single. In terms of their armed forces service, they were more likely to have served as a Private, or equivalent rank than those without an alcohol misuse diagnosis and to have had shorter service lengths. No other significant associations were found.

**Table 4.** Group comparisons (*χ*^2^ and *t*-tests) of those who screened positively for alcohol misuse (*n* = 59) and those who did not (*n* = 46).

Variable	Alcohol misuse	No alcohol misuse	OR	95% CI	*t* (d.f.)
Single marital status, *n* (%)	43 (73)	25 (54)	2.26^*^	.998–5.11	–
Pre-service vulnerability, *n* (%)	43 (73)	28 (61)	1.73	.757–3.94	–
Private rank, *n* (%)	49 (83)	27 (59)	3.45^**^	1.40–8.47	–
Deployment experience, *n* (%)	40 (68)	24 (52)	1.93	.871–4.28	–
Unemployed prior to prison, *n* (%)	23 (39)	23 (50)	1.57	.718–3.41	–
Money problems prior to prison, *n* (%)	26 (65)	14 (35)	1.80	.800–4.05	–
Low social contact, *n* (%)	13 (22)	14 (30)	1.55	.642–3.73	
Age, years					
Mean (SD)	36 (10)	49 (15)	–	–	5.24^**^ (78)
Median (min–max)	35 (20–66)	50 (23–88)			
Length of service, years					
Mean (SD)	5 (4)	8 (7)	–	–	2.44^*^ (66)
Median (min–max)	4 (1–20)	6 (1–30)			

* *p* < .05

** *p* < .01.

In terms of drug use, 29 (28%) participants screened positively as having a possible or definite substance abuse problem (see Table 2). Of these, 9 (9%) reported that they were currently receiving treatment for drug use in prison; this represented 31% of the population in need. Table 5 shows that there were no significant associations between having screened positively for drug misuse and pre-service vulnerability to negative health outcomes or having experienced deployment during their service. However, they were more likely than those who screened negatively for a drug misuse diagnosis to be younger and to have served as a Private, or equivalent rank. They were also more likely to have served shorter service lengths and to report unemployment and limited social contact prior to entering prison.

**Table 5.** Group comparisons (*χ*^2^ and *t*-tests) of those who screened positively for drug misuse (*n* = 29) and those who did not (*n* = 76).

Variable	Drug misuse	No drug misuse	OR	95% CI	*t* (d.f.)
Single marital status, *n* (%)	24 (83)	44 (58)	3.49^*^	1.20–10.13	–
Pre-service vulnerability, *n* (%)	22 (76)	49 (65)	1.73	.655–4.58	–
Private rank, *n* (%)	25 (86)	51 (67)	3.06^*^	.962–9.76	–
Deployment experience, *n* (%)	19 (66)	45 (59)	1.31	.536–3.19	–
Unemployed prior to prison, *n* (%)	19 (66)	27 (36)	.290^**^	.118–.712	–
Money problems prior to prison, *n* (%)	14 (35)	26 (65)	1.80	.753–4.28	–
Low social contact, *n* (%)	13 (45)	14 (18)	.278^**^	.109–.707	–
Age, years					
Mean (SD)	35 (9)	44 (15)	–	–	4.05^**^ (83)
Median (min–max)	35 (21–60)	44 (20–88)			
Length of service, years					
Mean (SD)	4 (3)	7 (6)	–	–	3.32^**^ (94)
Median (min–max)	3 (1–15)	6 (1–30)			

* *p* < .05

** *p* < .01.

Over half of those who screened as having CCMH problems additionally demonstrated dual symptomology, screening positively for an alcohol or drug misuse diagnosis (*n* = 23, 58%). Table 6 shows that those with dual symptomology were more likely to be younger and to have experienced deployment during their service. However, no other significant associations between those with dual symptomology, and those without, were found.

**Table 6.** Group comparisons (*χ*^2^ and *t*-tests) of those who dually screened for CCMH problems and substance misuse (*n* = 23) with those who screened positively for CCMH problems only (*n* = 17).

Variable	Dual screens	No dual screen	OR	95% CI	*t* (d.f.)
Single marital status, *n* (%)	20 (87)	12 (71)	2.78	.561–13.76	–
Pre-service vulnerability, *n* (%)	20 (87)	13 (77)	2.05	.393–10.70	–
Private rank, *n* (%)	18 (78)	14 (82)	.771	.157–3.79	–
Experience of deployment, *n* (%)	17 (74)	5 (29)	6.80**	1.68–27.52	–
Unemployed prior to prison, *n* (%)	10 (44)	11 (65)	2.38	.655–8.68	–
Money problems prior to prison, *n* (%)	16 (70)	8 (47)	2.57	.699–9.48	–
Low social contact, *n* (%)	10 (44)	7 (41)	.910	.256–3.24	–
Age, years					
Mean (SD)	36 (10.25)	45 (14.69)	–	–	2.30* (38)
Median (min–max)	34 (21–59)	43 (23–79)			
Length of service, years					
Mean (SD)	6 (4.44)	5 (3.81)	–	–	−1.04 (38)
Median (min–max)	4 (2–20)	3 (1–12)			

* *p* < .05

** *p* < .01.

## Discussion

This study describes the socio-demographic characteristics and mental health of a sample of male ex-armed forces personnel in six English prisons. Around a third of the sample screened as having a CCMH problem using the standardised measures. The most common diagnoses recorded in participants’ health care notes were for PTSD and depression. Over half of the sample screened positively for an alcohol misuse problem. There was also a high level of dual symptomology assessed with over half of those who screened positively for a CCMH problem also screening for an alcohol or drug misuse problem.

The ethnicity of the sample was predominantly white which is largely similar to that of the UK armed forces (95% vs. 93%, retrospectively) (Ministry of Defence [MoD], 2015) but demonstrates a higher proportion compared to the male prison population (93% vs. 73%) (Ministry of Justice [MoJ], 2016). Former service personnel in this study were older than their peers in the general prison population. This is in line with figures suggesting veterans in prison have an older age profile (Kelly, 2014; Statistics at MoD, 2010) than non-veteran prisoners (Berman & Dar, 2013).

One finding that shows a clear difference between the general prison population and ex-armed forces personnel in prison, was the number in employment prior to their current term. Thirty-two per cent of the general prison population report having been in employment in the month prior to entering prison (Prison Reform Trust, 2014), compared to 57% of ex-armed forces personnel in this study. Although this figure is based on self-report, personnel who have served in the armed forces are often credited as having a strong work ethic, which may be one explanation for this finding (House of Commons Defence Committee, 2013). Additionally, it may be that their service in the armed forces necessitates that they have less convictions overall than non-veteran prisoners.

The findings show that the overall prevalence of CMH problems amongst incarcerated ex-armed forces personnel are largely comparable to rates found within the general prison population (Singleton et al., 1998). However there were differences regarding the types of disorder experienced and, contrary to the recent inspectorate report (HM Inspectorate of Prisons, 2014), PTSD and depression were the most common diagnoses recorded in the health care notes, as opposed to depression and anxiety. It has been suggested that we may reasonably expect the prevalence of PTSD amongst prison populations to be higher than in the general population (Ardino, 2012). Previous research has found prevalence rates of PTSD of between 4 and 21% amongst general prison populations in the United States, New Zealand, Canada and Australia (Goff, Rose, Rose, & Purves, 2007). The current study suggests a higher prevalence of PTSD compared to the rate found in the general prison population in England and Wales (16% vs. 8%, retrospectively) (Singleton et al., 1998). The rate of recorded PTSD in imprisoned ex-services in this study is also higher than the 4% prevalence rate of probable PTSD found in the sample of 9990 serving and ex-service personnel in the community (Fear et al., 2010). However, the use of different measures and cut-off scores when screening for PTSD make comparisons of prevalence between samples difficult (for examples see Rona et al., 2014 and Sundin et al., 2014).

With regard to pre and post-service vulnerabilities, those who screened positively for CCMH problems were more likely to be considered ‘vulnerable’ prior to their service in the armed forces. This highlights the role pre-enlistment factors may play in the later development of mental health problems, similar to the findings of Iversen et al. (2007). Interestingly, no association between CCMH problems and having experienced deployment was found; however, significant associations were found with being single and having had money problems and limited social contact, prior to prison. For former service personnel in prison with CCMH problems, this demonstrates the likelihood of a wider spectrum of social and inter-personal difficulties with potential links to transition difficulties post-armed forces service. Being more likely to report limited social contact also lends support to the findings of Hatch et al. (2013) although, in this study, no association was found between CCMH and marital status.

In line with previous research showing high rates of co-morbidity amongst prisoners (Brooker, Sirdifield, & Gojkovic, 2007; Singleton et al., 1998), over half of those who screened positively for CCMH problems in this study also screened as having an alcohol or drug misuse problem. These participants were more likely to be younger and to have experienced deployment during their service in the armed forces. This suggests that we cannot ignore the role of military experiences in the development of mental health and substance misuse problems amongst this group. In terms of drug misuse, although based on a non-verified sample, the recent inspectorate report suggested ex-armed forces personnel involved in the CJS were less likely to have a drug misuse diagnosis than the general offending population (HM Inspectorate of Prisons, 2014). This study concurs finding that less than a third of the sample screened positively on the drug misuse measure. Those who screened as having a drug misuse problem were more likely to report being unemployed prior to prison.

Over half of the sample screened positively for an alcohol misuse problem, similar to the high rates of hazardous drinking found amongst the general prison population (56% vs. 58% for male remand prisoners and 63% for male sentenced prisoners) (Singleton et al., 1998). However, a later systematic review of thirteen studies of substance abuse amongst 7563 prisoners, Fazel, Bains, and Doll (2006) found somewhat lower estimates of alcohol misuse for male prisoners ranging between 18 and 30%. The current study may therefore suggest higher rates of alcohol misuse problems amongst incarcerated male ex-armed forces personnel. Indeed, levels of alcohol misuse have been found to be significantly higher amongst armed forces personnel in comparison to the general population (Fear et al., 2010). Further, a recent survey of offenders found ex-armed forces personnel were more likely to have alcohol misuse problems (Kelly, 2014) (although like the HMIP report this study is limited as a consequence of the veteran status of offenders not being confirmed). The current study corroborates the finding of Fear et al. (2007) who found that factors linked to a higher likelihood of alcohol misuse, amongst UK armed forces personnel, included being single and holding a lower rank. However, contrary to the study by Fear et al. (2010) who found deployment was significantly associated with alcohol misuse, no significant association between deployment and alcohol misuse alone was found in the current study.

### Strengths and limitations

This study, to the authors’ knowledge, presents the largest examination of the mental health of incarcerated ex-armed forces personnel in England to date. However, the cross-sectional, exploratory, nature of the study design only enabled limited analyses and we recognise that the sample size is small and therefore the generalisability of the findings are limited. This study aimed to describe the mental health and substance misuse needs of ex-armed forces personnel in prison and explore the potential aetiology of these issues but the lack of a comparison group does limit the conclusions that can be drawn.

All participants had freely identified as having previously served in the armed forces. This may act as a limitation of the study as an unknowable number of ex-personnel may have chosen not to self-identify as such in prison and may have different mental health needs. This similarly applies to those veterans who declined to take part in the study. Nevertheless, a strength of the study is that all participants’ military service was verified. Further, the mental health needs of the sample were assessed via a variety of sources- using information taken from their health care records, self-report and standardised, reliable, mental health assessments, adding credence to the findings. One issue that should be considered when interpreting the findings of this study is the likely fluctuation of scores on the measures used to screen for mental health problems. The participants in this study were interviewed at varying times after coming into prison and their symptoms may have presented differently throughout their time in custody. Previous studies have shown symptoms of mental distress may be more intense during early days of custody (Andersen et al., 2002; Hassan et al., 2011). Administering these measures at the same time point, and at more than one time, for all participants would have strengthened the study.

### Implications

The routine identification of ex-armed forces personnel entering prison establishments in England and Wales is a relatively new process, and the exact size of this group has been debated (Napo, 2008; Statistics at MoD, 2010). Before this study very little was known about the mental health needs and substance misuse problems of incarcerated ex-armed forces personnel in prison in England; this study therefore acts as an important first step in enhancing this knowledge base. This study demonstrates that ex-armed forces personnel likely do not require different mental health care than the general prison population. CMH problems were the primary concern for this group, with PTSD being the most common recorded diagnosis in participants’ health care notes. Therefore, training and knowledge of PTSD in general, and combat-related PTSD specifically, is essential within the prison environment. Ex-armed forces personnel were also likely to present with complex needs due to high levels of dual symptomology. This is particularly important for services to acknowledge, given the high rate of alcohol misuse within the sample. Prison can provide an opportunity to provide interventions and engage this group in treatment, including through the gate support, to target this issue.

That those who screened as having an alcohol or drug misuse problem in this study were significantly more likely to have shorter service lengths, indicates a link with research that has suggested ESLs may be more at risk of difficulties post service (Buckman et al., 2012). This further highlights that more support for this group when leaving the armed forces may be warranted to increase the likelihood of a successful transition. Further, a significant association between those with comorbid symptomology and having experienced deployment could recommend to the armed forces that general screening of troops post-deployment would be beneficial. In terms of providing support to this group in prison, associations between CCMH problems and lower levels of social contact and money problems, and between drug misuse and unemployment, highlight the potential wider level of social care need amongst this group. This suggests that this group may need particular help and guidance making the transition back into the community following their release from prison and may benefit from focused resettlement support. Indeed, preparing for release and resettlement post-prison for this group is considered important by both service users and professionals (Wainwright, McDonnell, Lennox, Shaw, & Senior, 2016).

Previous research has suggested that ex-armed forces personnel may engage with treatment and support services differently (Gould, Greenberg, & Hetherton, 2007; Iversen et al., 2005) including a general reluctance to engage at all and a significant delay between symptoms being experienced and them not being reported to a clinician. There is some indication in the results of this study that support this assertion. Of those who screened positively for an alcohol or drug misuse problem, less than a quarter reported being in current treatment. Further, the results show that less than half of those who reported they had a mental health problem were receiving treatment at the time of the interview. Whilst these findings warrant further investigation, we are unable to report whether these participants had accessed support, or been in contact previously, with health care services in prison. Additionally, a fifth of participants had been identified as being at current risk for suicide and/or self-harm at some point during their prison term, prior to taking part in the study. This finding may lend support to that of the HMIP report (2014) that ex-armed forces personnel were more likely to be suicidal on entry into prison. Of course, as with the findings regarding contact with health care services, we are unable to determine at what point during their prison term this was.

## Conclusions

The mental health needs of ex-armed forces personnel in this study seem to be fundamentally similar to those of the general prison population. However, there is potentially a higher prevalence of PTSD and alcohol misuse amongst former armed service personnel that prison staff and managers should consider. Comparisons of groups revealed that to fully understand the aetiology of mental health and substance misuse problems amongst this population, we need to understand the role of multiple factors including pre-service vulnerabilities, military experiences and issues post-service, such as the transition from military to civilian life. The results of the study also imply wider social care needs of the sample meaning through the gate provision may be particularly important for this group. Taking into account the limitations of the study, research needs to build on the current findings of this exploratory study to further our understanding of the needs of this group, and how they can be met. Alongside this, we also need to explore any potential barriers to care, and the help-seeking behaviour of this group. This will help ensure that we can provide effective support to this group in prison, and on release.
